# Ruptured infectious intracranial aneurysm related to infective endocarditis: report of a successfully treated case

**DOI:** 10.1093/ehjcr/ytaf078

**Published:** 2025-02-14

**Authors:** Hongkun Qing, Weiteng Wang, Yun Teng, Jimei Chen, Xuhua Jian

**Affiliations:** Department of Cardiovascular Surgery, Guangdong Cardiovascular Institute, Guangdong Provincial People’s Hospital, Guangdong Academy of Medical Sciences, Southern Medical University, No. 106, Zhongshan 2nd Road, Guangzhou 510060, P.R. China; Guangdong Cardiovascular Institute, Guangdong Provincial People’s Hospital, Guangdong Academy of Medical Sciences, Southern Medical University, No. 106, Zhongshan 2nd Road, Guangzhou 510060, P.R. China; Beijing Key Laboratory of Preclinical Research and Evaluation for Cardiovascular Implant Materials, Animal Experimental Centre, National Centre for Cardiovascular Disease, Department of Cardiac Surgery, Fuwai Hospital, Chinese Academy of Medical Sciences and Peking Union Medical College, No. 167 North Lishi Road, Xicheng District, Beijing 100730, P.R. China; Department of Cardiovascular Surgery, Guangdong Cardiovascular Institute, Guangdong Provincial People’s Hospital, Guangdong Academy of Medical Sciences, Southern Medical University, No. 106, Zhongshan 2nd Road, Guangzhou 510060, P.R. China; Department of Cardiovascular Surgery, Guangdong Cardiovascular Institute, Guangdong Provincial People’s Hospital, Guangdong Academy of Medical Sciences, Southern Medical University, No. 106, Zhongshan 2nd Road, Guangzhou 510060, P.R. China; Department of Cardiovascular Surgery, Guangdong Cardiovascular Institute, Guangdong Provincial People’s Hospital, Guangdong Academy of Medical Sciences, Southern Medical University, No. 106, Zhongshan 2nd Road, Guangzhou 510060, P.R. China

**Keywords:** Infective endocarditis, Infectious intracranial aneurysm, Endovascular therapy, Case report

## Abstract

**Background:**

Intracranial infectious aneurysm (IIA) is a rare but highly dangerous complication of infective endocarditis (IE), associated with a significant risk of rupture, particularly when anticoagulation is involved. Managing IE patients with IIA poses a significant challenge for both cardiac surgeons and neurologists.

**Case summary:**

We report a complex case of IIA related to IE in a 9-year-old girl with a history of patent ductus arteriosus ligation and aortic valvuloplasty. The patient was admitted for recurrent fever over 3 months despite irregular antibiotic therapy. Transthoracic echocardiography identified IE with vegetations on the aortic valve, and cerebral magnetic resonance angiography revealed an IIA. The patient experienced a sudden intracranial haemorrhage while awaiting intervention, requiring emergency craniotomy with haematoma removal and aneurysm resection. After 4 weeks of neurological rehabilitation and antimicrobial therapy, she recovered well without major neurological impairment. However, a recurrent IIA with haemorrhage was detected at the previously operated site. Following a multidisciplinary discussion, a one-stage procedure combining transcatheter aneurysm embolization and mechanical aortic valve replacement was performed successfully. Three years post-discharge, the patient maintained good cardiac and neurological function.

**Discussion:**

Managing IIA in patients with IE poses significant challenges due to the limited availability of high-level evidence. Endovascular therapy presents a promising approach, given its minimally invasive nature and the ability to expedite the initiation of anticoagulation therapy. This strategy could facilitate earlier cardiac surgery and potentially improve outcomes in critically ill patients.

**Learning points**

- Endocarditis patients should be routinely screened for neurological complications, particularly infectious intracranial aneurysms, and those with such aneurysms are generally advised to undergo cerebral procedures before cardiac surgery.
- Endovascular therapy is a preferred choice for patients with an indication for early cardiac surgery.

## Introduction

Intracranial infectious aneurysm (IIA) is a rare but lethal complication of infective endocarditis (IE), with high a mortality rate when ruptured.^1,2^ While IE patients are typically in critical condition with acute valvular dysfunction necessitating early surgery, the use of cardiopulmonary bypass and anticoagulation carries the risk of catastrophic intracranial haemorrhage (ICH) when IIA is present.^3^ The management of IIA related to IE remains a dilemma for cardiac surgeons and neurologists.

## Summary figure

**Figure:**
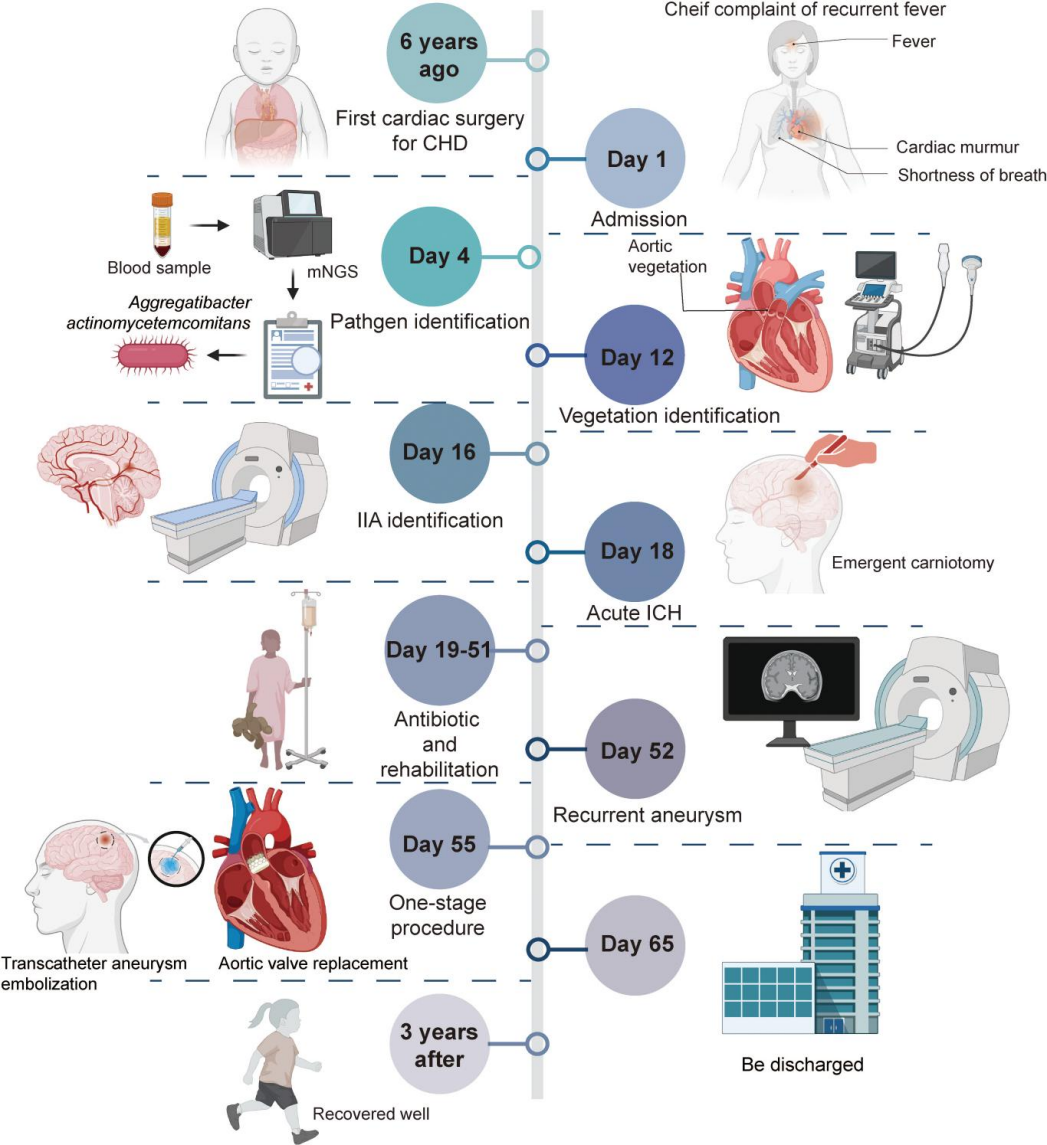
CHD, congenital heart disease; mNGS, metagenomic next-generation sequencing; IIA, intracranial infectious aneurysm; ICH, intracranial haemorrhage. The summary figure was created with Biorender.com.

## Case presentation

A 9-year-old girl was admitted to our hospital with recurrent fever over 3 months despite receiving intermittent antibiotic therapy at a local hospital. She had a history of patent ductus arteriosus ligation and aortic valvuloplasty at age 3. Routine follow-up echocardiography indicated progressive aortic regurgitation, with the latest scan before admission showing severe aortic regurgitation accompanied by left ventricular dilation. However, the patient remained asymptomatic.

**Figure 1:**
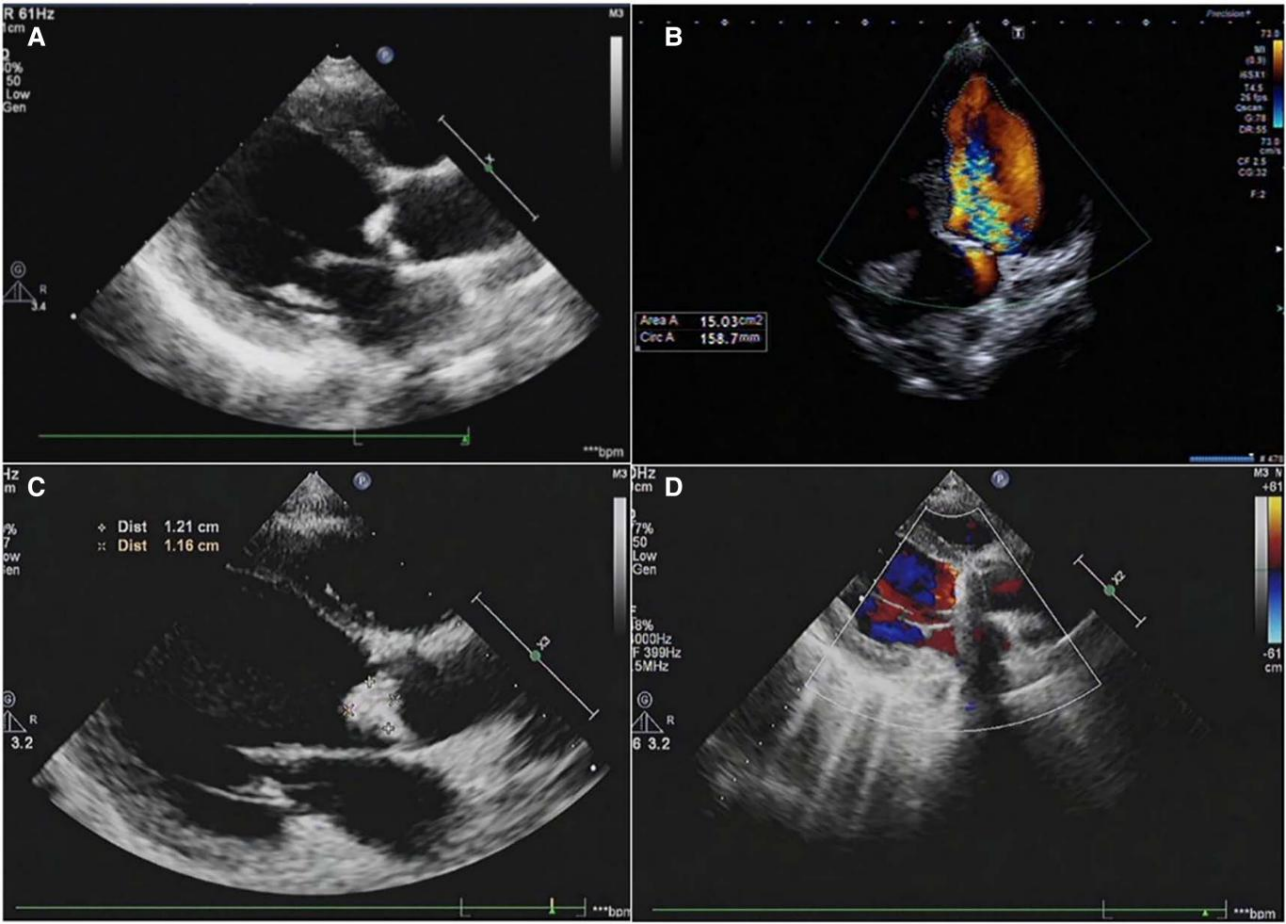
Transthoracic echocardiography findings: (*A*) at admission, demonstrating aortic vegetation; (*B*) at admission, revealing severe aortic regurgitation; (*C*) 1 month post-intracranial haemorrhage, displaying enlarging vegetation; and (*D*) prior to discharge, showing the implanted aortic prosthesis.

A detailed workup was carried out to clarify the cause of fever. While blood culture results were negative, mNGS on blood samples identified *Aggregatibacter actinomycetemcomitans*, a gram-negative bacillus commonly found in oral flora, also a member of the HACEK group (*Haemophilus, Aggregatibacter, Cardiobacterium, Eikenella,* and *Kingella*)—a significant cause of blood culture-negative IE. Additionally, transthoracic echocardiology (TTE) revealed vegetations on the aortic valve, with the largest measured 8 mm in length, leading to the diagnosis of IE (*Figure 1A* and *B*). Empirical antimicrobial therapy with intravenous ceftriaxone 80 mg/kg/day was initiated, and dental caries were removed as a potential source of infection.

**Figure 2:**
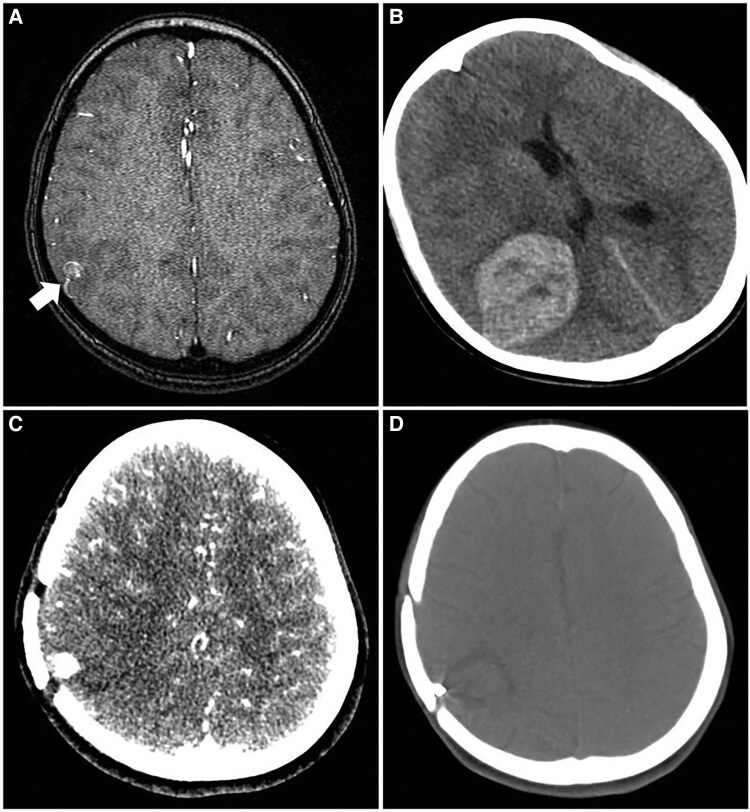
Index cerebral magnetic resonance angiography on admission (*A*, intracranial infectious aneurysm indicated by white arrow); computed tomographic angiography performed at the time of intracranial haemorrhaging (*B*), 1 month after craniotomy (*C*) and before discharge (*D*), respectively.

As part of routine screening for neurological complications, a cerebral magnetic resonance angiography (MRA) revealed the presence of an IIA on the right parietal lobe, measuring 8 × 6 mm (*Figure 2A*). Unfortunately, the patient experienced an acute ICH 2 days after the examination, before any intervention could take place. An emergency cerebral CT scan confirmed haemorrhage on the right parietal-temporal lobe, measuring up to 48 × 40 mm (*Figure 2B*), resulting from the rupture of the IIA. The patient was promptly referred to neurosurgeons, and an emergency craniotomy was performed for haematoma removal and aneurysm resection.

The patient experienced a full recovery without major neurological impairment after 4 weeks of neurological rehabilitation and antimicrobial therapy. As follow-up TTE revealed significant enlargement of the aortic valvular vegetation, measuring over 10 mm in length (*Figure 1C*), cardiac surgery was rescheduled due to the lack of response to antimicrobial therapy and the increasing size of the vegetation, which posed a high risk of embolism.

However, a repeat cerebral computed tomography angiography (CTA) revealed a recurrent aneurysm (7 × 7 mm) with haemorrhage at the previously operated site (*Figure 2C*), adding further complexity to the condition. Despite the indication for early surgery due to enlarging vegetation and severe aortic regurgitation, the recurrent IIA and concurrent haemorrhage posed a significant challenge to performing cardiac surgery.

**Figure 3:**
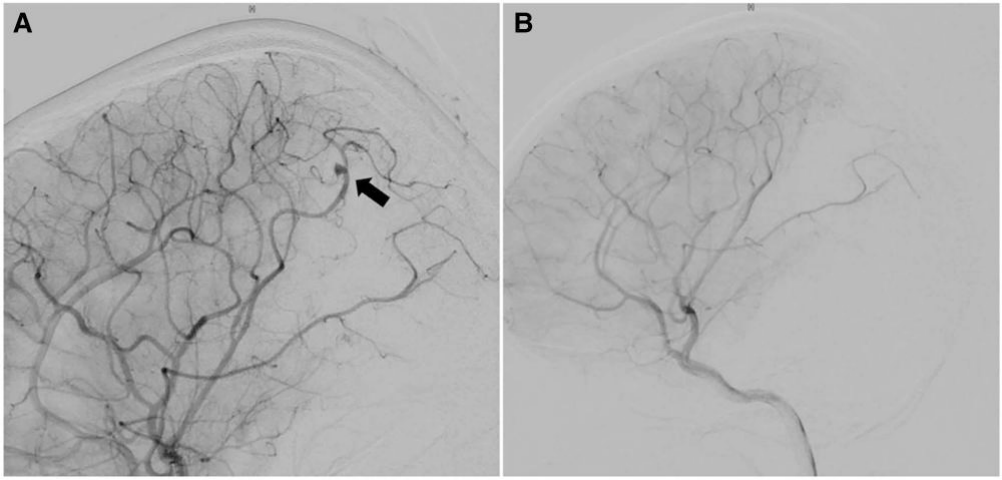
Digital subtraction angiography before (*A*, aneurysm indicated by black arrow), and after (*B*) the recurrent aneurysm has been embolized.

Following a multidisciplinary consultation, a consensus was reached to perform a one-stage procedure. This operation was carried out in a hybrid theatre and involved the combination of transcatheter aneurysm embolization (*Figure 3A* and *B*) with sequential aortic vegetation removal and mechanical valve replacement. The procedure proved to be a resounding success, and postoperative recovery progressed without complications. Postoperative work-up showed good aortic prosthesis function (*Figure 1D*) and complete isolation of the aneurysm without further haemorrhage before discharge (*Figure 2D*). Although both preoperative and postoperative blood cultures were negative, antimicrobial therapy was continued for another 4 weeks following cardiac surgery. Follow-up assessments indicated that the patient maintained good cardiac and neurological function 3 years after discharge.

## Discussion

IIAs result from the infection of arterial walls, most commonly caused by bacterial or fungal organisms. These aneurysms typically feature thin walls and fragility, making them more susceptible to rupture compared to berry aneurysms.^4^ This heightened vulnerability results in an exceedingly poor prognosis. Mortality rates for unruptured IIAs range from 10% to 30%, with the risk escalating to as high as 80% once rupture occurs.^5^

Infective endocarditis is the predominant cause of IIA, contributing to approximately 70% of all IIA cases.^2^ The reported incidence of IIA in IE patients is only 1%–9%, but this may be underestimated.^1,2,6,7^ The true incidence is probably higher since IIAs are often clinically silent prior to rupture and may resolve with antibiotic therapy. Nevertheless, given the high mortality of IIA, it should be routinely screened for in all IE patients. Enhanced CTA or MRA with contrast is of vital significance because IIA may not be detected by plain scans. Digital subtraction angiography remains the gold standard for IIA detection, and therefore should be considered when IIA is highly suspected but CTA or MRA are negative.^6,8^

Due to the relatively low incidence of IIA, relevant reports are scarce, mostly case reports or retrospective studies with a small number of cases. High-level evidence is missing. The management of IIA remains controversial. Antimicrobial therapy with close monitoring is mostly accepted as the initial treatment for unruptured IIA. However, in the study by Rice *et al*.^9^, only 28% of IIAs resolved or regressed under antimicrobial therapy alone, with a median resolution time of up to 36 days. This suggests that even with extended antibiotic dosing, the efficacy of antibiotics for IIA is limited. Furthermore, Alawieh *et al*.^2^ found that patients treated with antibiotics alone have a higher risk of treatment failure, often requiring salvage surgical or endovascular intervention. Therefore, a more aggressive strategy is advocated by some authors.^2,3,8^

Considering that anticoagulation is required during and after cardiac surgery, which could induce IIA rupture, IIAs should generally be secured preoperatively. Alawieh *et al*.^3^ recommend an interval of 4 weeks for cardiac surgery if the patient receives antimicrobial therapy alone, only after the IIA has resolved or regressed significantly. Typically, 2–3 weeks are needed after a craniotomy.^10^ However, IE patients are often in critical condition with acute heart failure; hence general anaesthesia and craniotomy carry high risks before cardiac surgery is performed. Compared to antibiotics alone or surgical intervention, endovascular therapy could be a preferred choice due to several advantages, including the use of local anaesthesia, potential for a short anaesthetic time, and minimal invasion.^2,4,11–14^ Most importantly, anticoagulation therapy can be initiated immediately after the procedure, allowing for the acceleration of cardiac surgery, even enabling simultaneous performance, as demonstrated by this case. This approach could be particularly beneficial for patients requiring urgent or emergency cardiac surgery. Craniotomy should be reserved for ruptured IIA with mass effect or those who failed transcatheter therapy, though.

There is a lack of reliable randomized controlled trial or large retrospective cohort data regarding the interval between IIA rupture and cardiac surgery. While a delay of at least 1 month for cardiac surgery following ICH is typically recommended by guidelines,^6^ our previous research has demonstrated the feasibility of performing early cardiac surgery once ruptured IIAs have been secured.^14^ Thus, it may be appropriate to manage ICH resulting from ruptured IIAs in a manner similar to unruptured IIAs, diverging from the general management principles of ICH.

## Conclusion

In summary, for patients with IIA related to IE, a more aggressive intervention may be deemed reasonable to avoid ICH events and provide a relatively safe condition for those with an indication for early cardiac surgery. Endovascular therapy may emerge as a preferred option, given its minimally invasive nature and the capability to expedite anticoagulation therapy.

## Data Availability

The data underlying this article will be shared on reasonable request to the corresponding author.

## Lead author biography

Qing H earned his MD from Peking University and is currently a cardiac surgeon at Guangdong Provincial People’s Hospital in Guangzhou, China. With training in adult cardiac surgery, paediatric cardiac surgery, and minimally invasive cardiac surgery, he has developed a particular interest in the multidisciplinary management of infective endocarditis.
